# Home monitoring with connected mobile devices for asthma attack prediction with machine learning

**DOI:** 10.1038/s41597-023-02241-9

**Published:** 2023-06-08

**Authors:** Kevin C. H. Tsang, Hilary Pinnock, Andrew M. Wilson, Dario Salvi, Syed Ahmar Shah

**Affiliations:** 1 Asthma UK Centre for Applied Research, Usher Institute, University of Edinburgh, Edinburgh, UK; 2 Medical Informatics, Usher Institute, University of Edinburgh, Edinburgh, UK; 3 Norwich Medical School, University of East Anglia, Norwich, UK; 4 Norwich University Hospital Foundation Trust, Colney Lane, Norwich, UK; 5 Internet of Things and People Research Centre, Malmö University, Malmö, Sweden

**Keywords:** Prognosis, Information technology

## Abstract

Monitoring asthma is essential for self-management. However, traditional monitoring methods require high levels of active engagement, and some patients may find this tedious. Passive monitoring with mobile-health devices, especially when combined with machine-learning, provides an avenue to reduce management burden. Data for developing machine-learning algorithms are scarce, and gathering new data is expensive. A few datasets, such as the Asthma Mobile Health Study, are publicly available, but they only consist of self-reported diaries and lack any objective and passively collected data. To fill this gap, we carried out a 2-phase, 7-month AAMOS-00 observational study to monitor asthma using three smart-monitoring devices (smart-peak-flow-meter/smart-inhaler/smartwatch), and daily symptom questionnaires. Combined with localised weather, pollen, and air-quality reports, we collected a rich longitudinal dataset to explore the feasibility of passive monitoring and asthma attack prediction. This valuable anonymised dataset for phase-2 of the study (device monitoring) has been made publicly available. Between June-2021 and June-2022, in the midst of UK’s COVID-19 lockdowns, 22 participants across the UK provided 2,054 unique patient-days of data.

## Background

Asthma is a long-term condition that affecting around 5.4 million people in the UK and its impact on daily life can vary from day-to-day^1^. Since there is no known cure for asthma, self-management is a key part of patient care; this involves detecting deterioration and taking appropriate action to maintain control and prevent the threatened attack^2^. Traditional self-management action plans use symptom scores, sometimes supplemented by peak expiratory flow measurements, to determine a patient’s asthma condition^3–5^. Keeping track of relief inhaler usage can also help measure asthma control^6^.

However, patients may regard this level of monitoring as tedious as it involves high levels of active engagement on their part. This is especially true when they are feeling well, because traditional monitoring is active and may yield repetitive readings. One method of alleviating some management burden is to reduce the manual labour associated with monitoring of asthma condition^7^. Passive monitoring (i.e. collecting data with minimum active user engagement), such as wearing a smartwatch, can provide an avenue to reducing the burden of monitoring if it is correctly used to give timely alerts^8^.

Three recent studies that have investigated the use of mHealth for asthma management were the Asthma Mobile Health Study (AMHS)^9,10^, myAirCoach^11^, and Biomedical REAl-Time Health Evaluation (BREATHE)^12^. The AMHS is the largest mHealth study for asthma conducted to date, containing large amounts of cross-sectional and longitudinal data. However, apart from the possibility of linking location data with historical weather reports, the dataset did not include any additional objective data (e.g., data from wearables, peak flow meters, or smart inhalers). To our knowledge, the dataset from myAirCoach and BREATHE are not publicly available and they have not yet been used to test any machine learning-based algorithms for asthma attack prediction^12–14^.

The AAMOS-00 observational study was carried out with the aim to provide a rich multi-dimensional dataset to develop better asthma attack prediction algorithms^15^. It combined market-available devices and application programming interfaces (APIs) to investigate the feasibility of a single mHealth system which pulls together data from sources available to asthma patients. Furthermore, the study included objective data sources (that can be collected passively) to complement current methods in asthma monitoring for self-management.

## Methods

### Data summary

A total of 22 patients participated in phase 2 of the study. Across 12 months, between 24^th^ June 2021 and 2^nd^ June 2022, 2,054 patient-days of data in phase 2. All participants of phase 2 agreed to share their anonymised data^16^. The average retention was 123 days (67%) in phase 2. The participants in phase 2 were mostly female (77%), average age of 40 years, mostly white (95%), and most (95%) had experienced an asthma attack in the past 12 months^16^ (see Table 1).

A course of oral corticosteroids (OCS) for an asthma attack in the past 12 months was an inclusion criterion for the study. This was asked twice, once before accessing the consent forms, and once at the start of the AAMOS-00 study (reported in Table 1). A potential explanation for observing one patient who did not have a course of OCS in the past 12 months, was that the patient had a course of OCS nearly a full 12 months ago, then had some delay with downloading Mobistudy and starting data collection. Thus, the number of patients who had a course of OCS for an asthma attack in the past 12 months was not 100%.

**Fig. 1:**
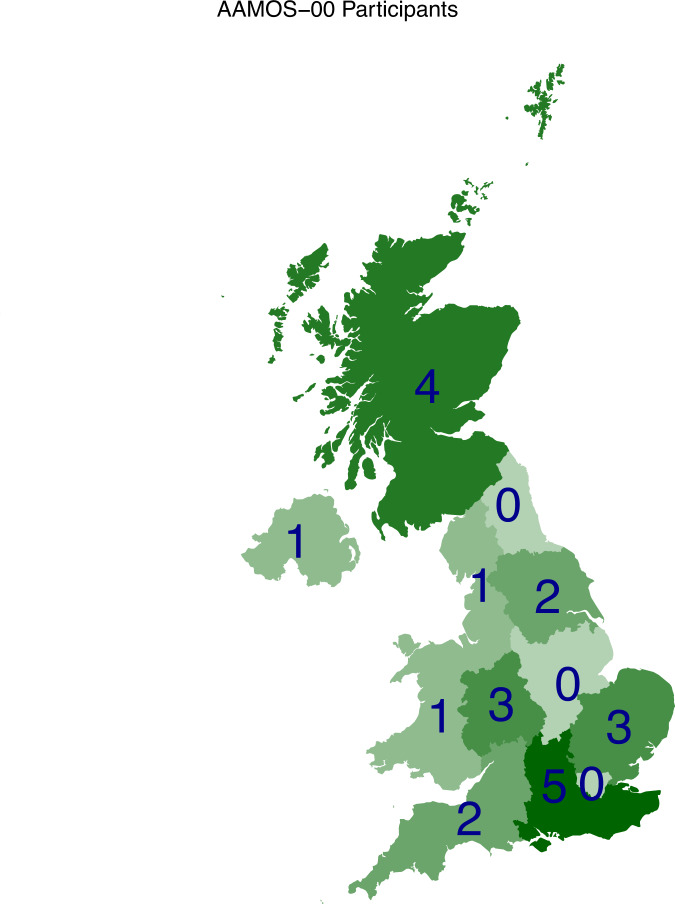
AAMOS-00 participants locations. Participants were located across all four nations of the UK.

Participants were located across all four nations of the UK (England, Northern Ireland, Scotland, and Wales), with a majority (73%) from England^16^ (see Fig. 1).

**Fig. 2:**
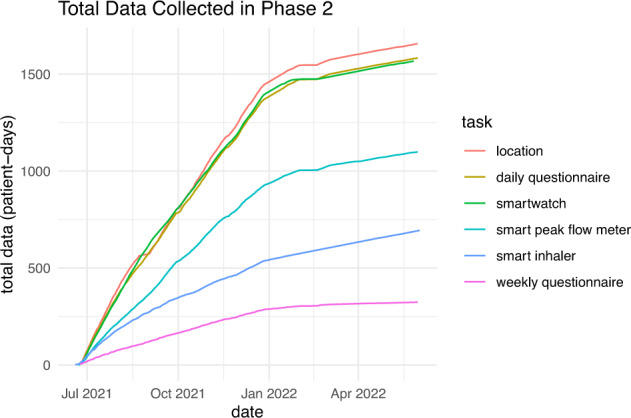
Total data collected.

In phase 2, a total of 1,583 daily questionnaires^16^ and 324 weekly questionnaires^16^ were collected. There were 694 patient-days of smart inhaler^16^, 1,567 patient-days of smartwatch data^16^, 1,099 patient-days of peak expiratory flow (PEF) recordings via the smart peak flow meter^16^, and 1,657 patient-days of sent locations^16^ (see Fig. 2). The total patient-days of relief puffs was relatively low since patients who spent a day without using the relief inhaler did not add to this count.

### Study design

The AAMOS-00 observational study conducted data collection over 14 months from April 2021 to June 2022. The study had two phases.

Phase 1 was one month of daily and weekly questionnaire monitoring, which was used to select participants who were likely to adhere to monitoring for a long duration in phase 2. The seven-item daily questionnaire combined the AMHS^9^ daily questionnaire with the Royal College of Physicians “3 Questions” (RCP3)^17^ questions to measure daily asthma control, triggers encountered, and medication usage (Supplementary Information). The AAMOS-00 study’s 11-item weekly questionnaire had incorporated aspects of the RCP3^17^, Asthma Control Questionnaire (ACQ)^4^, and AMHS^9^ weekly questionnaire to capture the asthma control and symptoms displayed in the past week, and any unscheduled care (Supplementary Information).

**Fig. 3:**
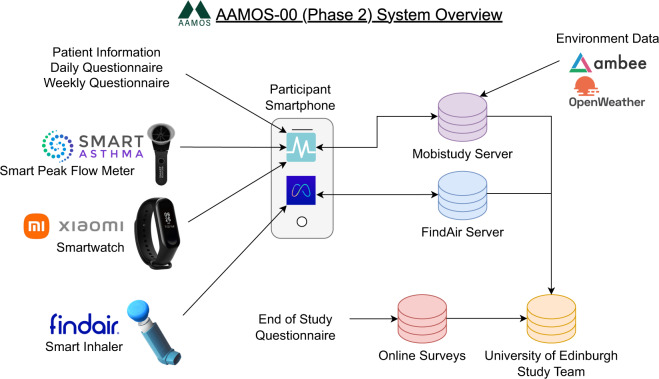
AAMOS-00 system overview. Centred around the participant’s own smartphone, three smart monitoring devices (smart peak flow meter, smartwatch, smart inhaler) collected objective data and two API services provided information about local environment (weather, air quality, pollen) based on the participant’s location.

Phase 2 was six months of smart device monitoring, where participants received three smart monitoring devices (smartwatch, smart peak flow meter, and smart inhaler) to collect data daily in addition to continuing daily questionnaires. Furthermore, daily location was used to link with weather, air quality, and pollen reports in the local area. The data collection was carried out via the Mobistudy app, which integrated all data collection apart from the FindAir smart inhaler (see the “Mobile Monitoring Technology” section for more details about the implementation and Fig. 3 for an overview of the system architecture).

Study participants of phase 2 had a total of four daily tasks (daily questionnaire, send location, morning and evening peak flow reading), two weekly tasks (smartwatch data upload and weekly questionnaire), and a passive monitoring task (smart relief inhaler usage).

At the end of phase 2, we asked participants to complete a questionnaire about the acceptability and usability of the system (Supplementary Information), to assess the current implementation and help future development. The three-part questionnaire used three validated questionnaires: the System Usability Scale (SUS)^18^ assessed usability, the mHealth Technology Engagement Index (mTEI)^19^ assessed personal motivation to use technology for self-management, and the User version of Mobile Application Rating Scale (uMARS)^20^ assessed app quality and perceived impact. We adapted some questions to better reflect the AAMOS-00 study system.

### Recruitment

Participants across the UK were recruited via social media, invitation letters from Norfolk and Norwich University Hospitals (NNUH), and email invitations. Participants were adults with asthma who had experienced an asthma attack in the past 12 months (definition: ATS/ERS Task Force 2009)^21^ and were prescribed with a pressurised metered dose relief inhaler that was compatible with FindAir ONE.

Social media recruitment consisted of disseminating invitations to the public via Twitter and Facebook via the Asthma + Lung UK and Asthma UK Centre for Applied Research (AUKCAR) accounts, which total around 175,000 followers. The Norfolk and Norwich University Hospital helped identify potentially eligible patients for the study and had sent them invitation letters. Email invitations were sent via the Asthma UKs Research and Policy Volunteers Bulletin, which is a channel to circulate research opportunities conducted by the Asthma + Lung UK to volunteers. Patients who were interested in joining the study were directed to the recruitment website, where they found the participant information sheets and the online consent form hosted on Online Surveys.

**Fig. 4:**
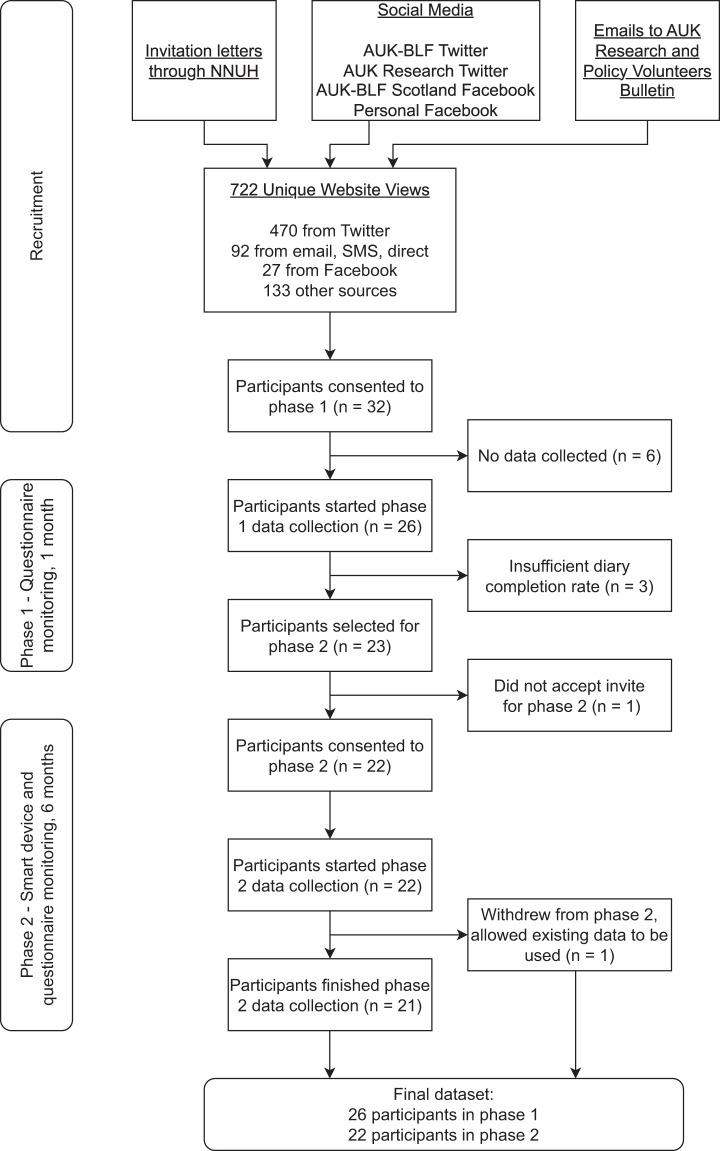
AAMOS-00 participants engagement. Phase 1 was one month of daily questionnaire monitoring. Phase 2 was six months of smart device and questionnaire monitoring.

Over the 10-month recruitment period from 15^th^ February 2021 to 1^st^ December 2021, 32 participants were recruited to phase 1 of the study (see Fig. 4). After one-month of data collection with daily questionnaires, 23 participants who had completed at least half of the requested daily questionnaires (14 of 28 days) were selected and invited to join the device monitoring portion of the study (phase 2). One participant declined the invitation. Twenty-two participants collected data for six months and one participant pulled out of the study during phase 2, citing frustration with the technology.

### Mobile monitoring technology

#### Mobistudy

Mobistudy^22^ is an open-source platform for facilitating mobile-based studies, it is managed by Malmö University, Sweden. The platform has three key components: a mobile app for participants (available for Android and iOS), a representational state transfer (REST) API server, and an online web portal for researchers to view the data in real time. The platform supports multiple studies and participants of the AAMOS-00 study were given a study invite code to join the AAMOS-00 study within Mobistudy.

The Mobistudy mobile app was central to data collection where each daily and weekly assessment (questionnaires, peak flow measurement, smartwatch data upload, and sending location) appeared as an individual task of the home page on the participant’s app.

#### Smart Asthma Smart Peak Flow Meter

The Smart Peak Flow Meter by Smart Asthma (www.smartasthma.com) is an affordable (£34.99) mHealth peak flow meter that measures peak flow (PEF) with the help of the processing in the smartphone. There are two modes of connection between the device and the smartphone, by direct audio jack connection or by a Bluetooth adapter. The device is a Class 2a medically certified device and has been tested and validated against a pulmonary waveform generator, deemed to pose a low to medium risk to patients and thus complies with the UK’s and EU’s safety and performance standards^23–25^. The device connected directly to the Mobistudy app, and the signal was translated into peak flow reading via the integrated library provided by Smart Asthma.

#### Xiaomi Miband3 smartwatch

The MiBand3 by Xiaomi (www.mi.com) is an affordable (£25.00) smartwatch that can be used to monitor activity and heart rate. The CE (Conformité Européenne) marked device is lightweight and includes a touch screen where navigate to different screens such as total steps today, battery, exercise mode, and current heart rate. Four minute-by-minute signals were collected with the MiBand3, heart rate reading, activity type, activity intensity, and total steps. The data upload from the smartwatch used a Bluetooth connection to Mobistudy and an integrated library.

#### FindAir ONE smart inhaler

The FindAir ONE by FindAir (www.findair.eu) is a smart inhaler (also known as a Bluetooth cap) for pressurized metered-dose inhalers (pMDI) inhalers, to track when the inhaler is used, priced at €59.00 per year. The CE marked device has a battery life of 12 months after first use and is un-rechargeable. Once attached to the inhaler, the smart inhaler automatically logs actuations when the inhaler is used. When the device is connected to the smartphone via Bluetooth, the stored data is transferred to the smartphone and then FindAir’s server. We used a secure REST API connection to transfer data between the FindAir server to the study’s server (see Fig. 3).

#### Open Weather Maps and Ambee

Based on the location of the participant when completing the location task, the local weather, air quality, and pollen count were fetched using Open Weather Maps’^26^ and Ambee’s^27^ APIs (see Fig. 3). The information included weather, temperature, humidity, cloud cover, wind, air quality index (AQI)^28^, and grass, tree, and weed pollen count.

#### Online Surveys

The study consent forms and the exit questionnaire about usability and acceptability were hosted on Online Surveys (https://www.onlinesurveys.ac.uk/), which is an online service where participants can visit a webpage to complete the questionnaires and the data is then securely held by their servers. Afterwards, the responses were transferred to the study’s servers.

#### Data anonymisation

The directly identifiable information fields^29^ were removed or replaced. These included name, dates (including date of birth and date of data entry), location, height, weight, medication used, and user key.

- The names of participants were removed.
- The age in years was calculated at the end date of the study, which replaced the participant’s date of birth information. Furthermore, the age was reduced in granularity via the use of age ranges.
- Likewise, only the age range of the age of asthma diagnosis was made available. The ranges were early childhood (0 to 6 years old), late childhood (7 to 11 years old), adolescence (12 to 18 years old), and late onset (19 + years old)^30^.
- The daily locations of participants were not made available, but the local environmental data (weather, air quality, and pollen count) collected during the study were made available. A single location of participants at the UK region level was not identifiable and was made available. The information would be sufficient to link localised historic weather data.
- Body mass index (BMI), calculated from height and weight, and theoretical maximum PEF, calculated from height, age, and sex, were known risk factors of asthma attacks^3,4^ and important measures. The BMI range and theoretical maximum PEF rounded to the nearest 5 were made available.
- The list of medication used by patients were removed.
- The participant user keys were replaced with a new random number between 100 and 999.
- All dates of data entry in the dataset were removed. The dates of data entry were transformed to the number of days after each participant started phase 2.

Patient sex and race were made available, because there are known sex and ethnic differences in asthma^31–33^. They were indirectly identifiable information and considered to have a low risk of deanonymisation.

#### Data pre-processing

The published dataset was produced by combining raw JSON, CSV, and XSLX files from the aforementioned data sources. All the data pre-processing was conducted using R (v4.2.1)^34^ and the following packages: fuzzyjoin^35^, gtools^36^, janitor^37^, jsonlite^38^, lubridate^39^, plyr^40^, PostcodesioR^41^, qdapTools^42^, rapportools^43^, readxl^44^, tidyverse^45^, zoo^46^.

#### Ethics

Ethics approval was provided by the East of England - Cambridge Central Research Ethics Committee. IRAS (Integrated Research Application System) project ID: 285505 with governance approval from ACCORD (Academic and Clinical Central Office for Research and Development), project number: AC20145. The study sponsor is ACCORD, the University of Edinburgh. The anonymised data has been made available with the consent of each participant.

#### Limitations

A major limitation of the AAMOS-00 study was the narrow inclusion criteria, which selected asthma patients who had an interest in monitoring and had experienced a severe asthma attack in the past 12 months. Although the dataset contains limited patients, there are over 2000 unique patient days of longitudinal multi-dimensional data. Speaking with patient and public involvement (PPI) members, we believed the average retention to daily monitoring in the general population would be substantially lower than what we observed in this study. However, the average retention in phase 2 was 123 days, which included daily tasks, this was much longer than our initial estimate.

Due to the technical implementation of using a mobile phone app to accommodate the general population, the passive monitoring devices of location and smartwatch require daily and weekly active engagement with the app. This meant that this study could not explore the potential of completely passive monitoring devices that require no user intervention. Furthermore, the technical issues encountered by some patients could have affected the adherence to monitoring with the smart devices.

The AAMOS-00 study was conducted over several periods of national lockdowns in the UK due to the COVID-19 pandemic^47^. During this unique time period, the general public had spent more time indoors, had facemask wearing, kept social distancing, and had reduced road traffic^48^, which reduced the exposure to some triggers such as virus, pollutants, and outdoor allergens. The effect of lockdowns was a substantial reduction in asthma attack rates^49^. Comparing this data with other dataset collected during times without lockdowns could provide further insights into how a drastic societal change has affected asthma.

## Data Records

Researchers are able to download the anonymised data from phase 2 of the AAMOS-00 study^16^ via Edinburgh DataStore (a digital repository of research data produced at the University of Edinburgh) (https://datashare.ed.ac.uk/handle/10283/4761). The dataset was designed for longitudinal analysis, linking the different signals by “user_key” and “date” would give the most holistic view of the patients. The date begins at 1 for each patient, this represents the first day of data entry in phase 2 for each patient. Each increment is a calendar day.

The dataset contains a separate file for each data signal. The list of files provided are as follows:

- Data Documentation – contains a information about the dataset and the study
- Data Dictionary – contains the data definition for all variables
- Daily Questionnaire Data – includes the responses to the daily questionnaire (including symptoms, medication use, triggers encountered)
- End of Study Questionnaire Data – includes the usability and acceptability score collected at the end of the study
- Environment Data – includes the daily weather, pollen, air pollution data
- Patient Information – includes the patient information (including age, sex, region) collected at the start of the study
- Smart Peak Flow Meter Data – includes twice-daily measurements of peak flow measured via the smart peak flow meter
- Smart Inhaler Data – includes relief inhaler usage information (timestamp and medication name) collected via smart inhaler
- Smartwatch Data 1 (data entries 1-1,000,000) – includes the minute-by-minute data from the smartwatch (including activity type, intensity, steps taken, heart rate), set one
- Smartwatch Data 2 (data entries 1,000,001–2,000,000) – set two of the smartwatch data, continues from set one
- Smartwatch Data 3 (data entries 2,000,001–2,101,829) – set three of the smartwatch data, continues from set two
- Weekly Questionnaire Data – includes the responses to the weekly questionnaire (including asthma control, symptoms, and any unscheduled care)

Further details about all the variables in each data file can be found in the Data Dictionary^16^ (Supplementary Table).

## Technical Validation

During the development of Mobistudy and the integration of external sensors, standard software engineering techniques for quality assurance were put in place, such as extending testing and, when feasible, automated tests. Sensors integrated algorithms to extract physiological measurements from raw data, either in their internal firmware (MiBand 3) or as software libraries (Smart Peak Flow meter), which were integrated into the app. All smartwatches underwent a software update, to ensure that the latest firmware version (v2.4.0.32) was installed.

Our research team, comprising clinicians, checked that all values from questionnaires and devices were clinically plausible. The smart peak flow meters provided readings within our expectation. Only two peak flow measurements were outside theoretical range of values (based on age, sex, and height)^50^. For both readings, a valid peak flow reading taken within minutes replaced the outlier value. From the smartwatch, the mean sleep duration per day was 7.6 hours (interquartile range of 3.1 hours), which was within the expected range. Also from the smartwatch, the mean heart rate was 82 BPM, which was high but within physiological range (normal resting heart rate is between 50 and 90 BPM)^51^.

**Fig. 5:**
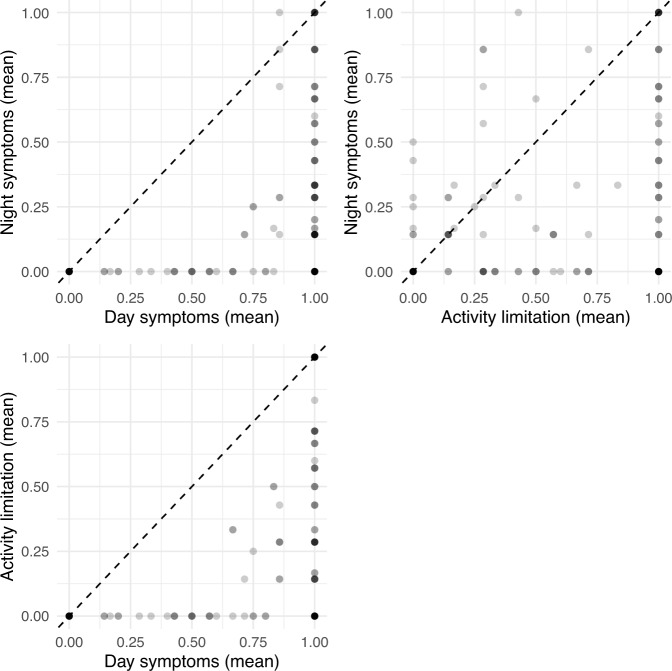
Comparison of daily answers to questions for asthma control averaged over a week (0 = no symptoms recorded on any day of the week, 1 = symptoms present on all days of the week). In line with previous understanding^52^, the day symptoms were almost always higher than night symptoms and activity limitation, whereas nocturnal symptoms and activity limitation were relatively independent.

The daily asthma control questions displayed expected characteristics. As reported by Pinnock *et al*.^52^, the day-time symptoms alone is less of an indication of poor control than nocturnal symptoms or activity limitation alone. The AAMOS-00 data supports the finding. In general, the day symptoms were almost always higher than night symptoms and activity limitation, whereas nocturnal symptoms and activity limitation were relatively independent (see Fig. 5). As a feature, this means that day symptoms were superseded by the information of night symptoms and activity limitation.

**Fig. 6:**
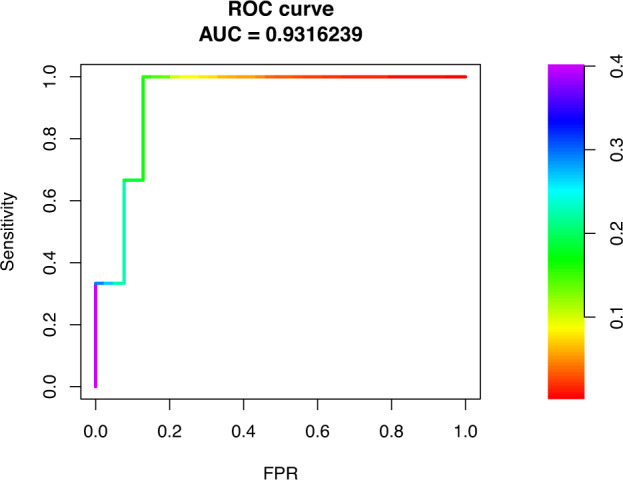
ROC curve of random forest classifier. AUC = 0.93 and AUPRC = 0.55 suggesting a strong signal was present. Data had 15 positive class and 159 negative class.

Reframing this data as binary classification problem, we attained a high area under the receiver operating characteristic (ROC) curve (AUC) and area under the precision-recall curve (AUPRC) to classify weeks where patients attended unscheduled asthma doctor appointments using daily data, suggesting the classifier performs well using this data. Data processing was used to extract the weekly mean value of daily data in the dataset (from the daily questionnaire, environment, smartwatch, smart peak flow meter, and smart inhaler). This formed a dataset with 15 observations in the positive class (attended unscheduled appointment during the week) and 159 observations in the negative class. Then using an 80%-20% training-test split, we trained a random forest classifier and achieved good performance (AUC = 0.93 and AUPRC = 0.55) (see Fig. 6).

## Usage Notes

The dataset is licensed under the Creative Commons Attribution 4.0 International Public License (CC BY 4.0).

To download the dataset, visit the DataShare page (https://datashare.ed.ac.uk/handle/10283/4761).

R scripts have been provided to assist the usage of this dataset, including joining data tables, data wrangling, and an example binary classification problem.

**Table 1:** AAMOS-00 phase 2 participant characteristics.

Characteristics	AAMOS-00 Phase 2 (n = 22)
**Sex**, n (%)	
Female	17 (77%)
Male	5 (23%)
**Age**, median (IQR)	40.2 years old (15.7 years old)
**BMI**, mean (SD)	27.7 kg/m^2^ (5.7 kg/m^2^)
**Race (White)**, n (%)	21 (95%)
**Smoker**, n (%)	
Never (<100 cigarettes)	17 (77%)
Previous	5 (23%)
**Had hospitalisations in past 12 months**, n (%)	6 (27%)
**Had a course of OCS for asthma attack in past 12 months**, n (%)	21 (95%)
**RCP3 in past month**, mean	2.4

Royal College of Physicians “3 Questions” (RCP3) score ranges from 0 to 3, 0 indicating good control, 1–3 indicating poor control^17^.

## Supplementary Information

AAMOS-00 Questionnaires

Supplementary Table

## Data Availability

The Mobistudy version 0.2.6 used in the AAMOS-00 study can be found at https://github.com/Mobistudy. The software to translate the smart peak flow meter signal into peak flow was integrated with Mobistudy using a Cordova plugin, the code can be found on GitHub: https://github.com/kevinchtsang/cordova-plugin-spf. The smartwatch was integrated into Mobistudy based on the open-source work by Volodymyr Shymanskyy (https://github.com/vshymanskyy/miband-js), José Rebelo, and Gadgetbridge (www.gadgetbridge.org). R scripts to illustrate joining the data tables and forming a binary classification problem can be found at https://github.com/kevinchtsang/AAMOS-00-Starter.
