# Supplementary material for: Home monitoring with connected mobile devices for asthma attack prediction with machine learning
Source: Sci Data. 2023 Jun 8;10:370. doi: 10.1038/s41597-023-02241-9 (PMC10248342; doi:10.1038/s41597-023-02241-9)
Supplement: Supplementary file 1 — AAMOS-00 Questionnaires [file 41597_2023_2241_MOESM1_ESM.pdf]

Eligibility – Phase 1 (Asked before joining the study)

1. Are you aged 18 and above?
    a. Yes
    b. No
2. Have you been diagnosed with asthma by a doctor?
    a. Yes
    b. No
3. Do you have an Android smartphone from 2016 onwards (e.g. Samsung Galaxy S7, Xiaomi Mi 5, Huawei P9) or iPhone 7 or later with Bluetooth?
    a. Yes
    b. No
4. Have you had at least one course of oral corticosteroids (Prednisone, Medrol, etc.), for an acute asthma attack, in the past 12 months?
    a. Yes
    b. No

If Yes to all 4 questions:

"Thank you for your interest in participating in our study. Please read the participant information sheet for more detail and proceed to (https://edinburgh.onlinesurveys.ac.uk/aamos-00-phase-1-consent) to join the study."

If No to any question:

"Thank you for your interest in participating in our study. Based on your responses, you are not eligible to take part.

You can find out more about your asthma at Asthma UK. See if you are eligible to join any other asthma research with the Asthma UK Centre for Applied Research. You could also read more about Kevin Tsang, the research lead for this study."

Eligibility – Phase 2 (Asked to people who have participated in phase 1)

1. Have you taken part in phase 1 and completed at least half the daily questionnaires in phase 1?
    a. Yes
    b. No
2. Are you prescribed with a pMDI (pressurised metered dose inhaler) ("L-shaped") relief inhalers ("blue puffer")? (See picture) Some common brands: (Ventolin, Salamol, Airomir, Fostair, Budiair)
    a. Yes
    b. No, I am not prescribed with a pMDI ("L-shaped") inhaler
    c. Not sure

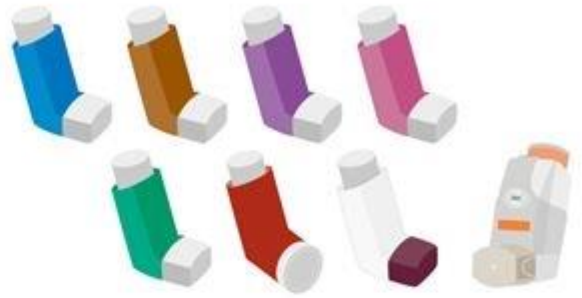

3. Do you intend to be in the UK for the majority of the next 6 months?
    a. Yes
    b. No

If Yes to all 3 questions:

"Thank you for your interest in participating in our study. Please read the participant information sheet for more detail and proceed to (https://edinburgh.onlinesurveys.ac.uk/aamos-00-phase-2-consent) to join the study."

If No to any question:

"Thank you for your interest in participating in our study. Based on your responses, you are not eligible to take part.

You can find out more about your asthma at Asthma UK. See if you are eligible to join any other asthma research with the Asthma UK Centre for Applied Research. You could also read more about Kevin Tsang, the research lead for this study."

If Not sure in question 2:

"If you're not sure about the inhaler device you're using please contact us with a picture of your relief inhaler. Email Kevin Tsang at k.c.h.tsang@sms.ed.ac.uk. You may return to this questionnaire later."

About you (Asked at the start of phase 1)

1. What is your year of birth?
    a. Number input
2. What is your sex?
    a. Male
    b. Female
3. What is your ethnic group? Choose one option that best describes your ethnic group or background

Asian/ Asian British

- Indian

- Pakistani
- Bangladeshi
- Chinese
- Any other Asian background, please describe

Black/African/Caribbean/Black British

- African
- Caribbean
- Any other Black /African/Caribbean background, please describe

Mixed/multiple ethnic groups

- White and Black Caribbean
- White and Black African
- White and Asian
- Any other Mixed/multiple ethnic background, please describe

White

- British/ English/ Northern Irish/ Scottish/ Welsh
- Irish
- Gypsy or Irish Traveller
- Any other White ethnic group, please describe

Other ethnic group

- Arab
- Any other ethnic group, please describe

4. What is your height?
    a. Select units (cm or inches)
    b. Number input
5. What is your weight
    a. Select units (kg or pounds)
    b. Number input
6. What is your smoking status?
    a. Never (<100 cigarettes in lifetime)
    b. Current
    c. Previous [go to question 7]
7. When did you stop smoking?
    a. Year input
8. On average, how many cigarettes do/did you smoke daily?
    a. Number input
9. How many years have you been smoking/did you smoke?
    a. Number input

Asthma specific (Asked at the start of phase 1)

1. Do you think of your asthma as:
    a. Very mild
    b. Mild
    c. Moderate
    d. Severe
    e. Very severe
2. What triggers your asthma? (Select all that apply)
    a. A cold
    b. Exercise
    c. Being more active than usual
    d. Strong smells
    e. Exhaust fumes
    f. House dust
    g. Dogs
    h. Cats
    i. Other furry/feathered animals
    j. Feathers
    k. Mould
    l. Pollen from trees grass or weeds
    m. Extreme heat
    n. Extreme cold
    o. Changes in weather
    p. Around the time of my period
    q. Poor air quality
    r. Someone smoking near me
    s. Foods
    t. Stress
    u. Feeling sad, angry, excited, tense
    v. Laughter
    w. I don't know what triggers my asthma
    x. Other (please specify)
3. Have you been hospitalised for asthma in the past 12 months?
    a. Yes
    b. No
4. Have you been to the A&E (emergency department) for asthma in the past 12 months?
    a. Yes
    b. No
5. Have you had a course of corticosteroids (Prednisone, etc.) for three days or more for an asthma attack in the past 12 months?
    a. Yes
    b. No
6. In the past month, have you had difficulty sleeping because of your asthma symptoms (including cough)?
    a. Yes
    b. No

7. In the past month, have you had your usual asthma symptoms during the day (cough, wheeze, chest tightness, or breathlessness)?
    a. Yes
    b. No
8. In the past month, has your asthma interfered with your usual activities (e.g. housework, work/school, etc.)?
    a. Yes
    b. No
9. What is your personal best peak flow (PEF) measurement? (Average personal best peak flow ranges between 300 and 700)
    a. Number input (Units: litres of air breathed out per minute, L/min)
    b. I am not sure
10. How old were you when first diagnosed with asthma, or when you had your first symptoms?
    a. Number input
11. How many different relief inhalers ("blue puffer") do you use on a regular basis?
    a. Number input

Daily Questionnaire (Asked daily in phase 1 and 2)

1. Have you had difficulty sleeping because of your asthma symptoms (including cough) in the past 24 hours?
    a. Yes
    b. No
2. Have you had your usual asthma symptoms during the day (cough, wheeze, chest tightness or breathlessness) in the past 24 hours?
    a. Yes
    b. No
3. Has your asthma interfered with your usual activities (for example housework, work or school) in the past 24 hours?
    a. Yes
    b. No
4. Did you use your asthma preventer inhaler in the past 24 hours?
    a. No, I did not take them
    b. Yes, my usual doses
    c. Yes, less than my usual doses
    d. Yes, more than my usual doses
    e. I'm not sure
5. How many puffs of your relief inhaler ("blue puffer") did you have in the past 24 hours?
    a. None
    b. 1 – 2
    c. 3 – 4
    d. 5 – 8
    e. 9 – 12
    f. 12 or more
6. Did you encounter any asthma triggers today? Select all that applies
    a. A cold
    b. Exercise or being more active than usual
    c. Strong smells / exhaust fumes
    d. Mould
    e. House dust / dust mites
    f. Feathers
    g. Dogs / cats / other furry / feathered animals
    h. Pollen from trees grass or weeds
    i. Extreme heat
    j. Extreme cold
    k. Changes in weather
    l. Poor air quality
    m. Someone smoking near me
    n. Foods
    o. Around the time of my period
    p. Feeling sad, angry, excited, tense, stressed
    q. Laughter
    r. I am not sure
    s. Other (please specify)

Weekly Questionnaire (Asked weekly in phase 1 and 2)

1. How many days over the last week have you had difficulty sleeping because of your asthma symptoms (including cough)?
    a. Number input 0 - 7
2. How many days over the last week have you had your usual asthma symptoms during the day (cough, wheeze, chest tightness or breathlessness)?
    a. Number input 0 - 7
3. How many days over the last week has your asthma interfered with your usual activities (e.g. housework, work, school, etc)?
    a. Number input 0 - 7
4. Which of the following statement best describes how often you had experienced a shortness of breath due to your asthma in the last seven days?
    a. I did not experience any shortness of breath due to my asthma
    b. I sometimes had a shortness of breath due to my asthma
    c. I had a moderate amount of shortness of breath due to my asthma
    d. I often had a shortness of breath due to my asthma
    e. I had a shortness of breath due to my asthma all the time
5. Which of the following statement best describes how often you had experienced wheezing due to your asthma in the last seven days?
    a. I did not wheeze due to my asthma
    b. I sometimes wheezed due to my asthma
    c. I had a moderate amount of wheezing due to my asthma
    d. I often wheezed due to my asthma
    e. I was wheezing all the time due to my asthma
6. Which of the following statement best describes your average daily total puffs of your relief inhaler ("blue puffer"), except for use before exercise, in the last seven days?
    a. I had not used my relief inhaler
    b. I had 1 – 2 puffs of my relief inhaler
    c. I had 3 – 4 puffs of my relief inhaler
    d. I had 5 – 8 puffs of my relief inhaler
    e. I had 9 – 12 puffs of my relief inhaler
    f. I had 12 or more puffs of my relief inhaler
7. Did you visit a general practice (GP) or asthma doctor, not including regular visits regarding your asthma in the past week? If so, please select which days
    a. Select day of week (multiple choice from 7 dates)
8. Were you hospitalised due to your asthma in the past week? If so, please select which days
    a. Select day of week (multiple choice from 7 dates)
9. Did you visit an emergency room (ER) for your asthma in the past week? If so, please select which days
    a. Select day of week (multiple choice from 7 dates)
10. Did you use systemic corticosteroids (Prednisone, etc.) in the past week?
    a. No
    b. Yes, but no more than usual
    c. Yes, more than usual
    d. I'm not sure
11. Please describe any other asthma symptoms you have experienced over the last week.
    a. Free text

Some of the questions were adapted from the questions listed in Supplementary Table 1 of the Asthma Mobile Health Study [PMID: 29786695], an open access article licensed under a Creative Commons Attribution 4.0 International License https://creativecommons.org/licenses/by/4.0/

Chan, Yu-Feng Yvonne, Brian M. Bot, Micol Zweig, Nicole Tignor, Weiping Ma, Christine Suver, Rafhael Cedeno et al. "The asthma mobile health study, smartphone data collected using ResearchKit." *Scientific data* 5, no. 1 (2018): 1-11.

End of Study Questionnaire (End of Phase 2)

Context: the "end of study questionnaire" for AAMOS-00 will be asked at the end of the 6-month phase 2, which will have given the chance for participants to use three smart monitoring devices (FindAir ONE smart inhaler, SRP smart peak flow meter, and XiaoMi MiBand3 smartwatch) and two mobile apps (Mobistudy and FindAir). The questionnaire aims to assess the usability and acceptance of the devices and apps. Also, to help future development of asthma management apps.

All answers "scale 1-5" refer to a 5-point Likert scale: (1) Strongly disagree; (2) Disagree; (3) Neither agree nor disagree; (4) Agree; (5) Strongly agree.

Questionnaire Introduction: In this questionnaire, we will ask about your experience and feedback from using the data collecting system in phase 2 of AAMOS-00 (including Mobistudy, smartwatch, smart peak flow meter, and smart inhaler and associated app) as a whole.

Section 1 – Usability [System usability scale (SUS)]

1. I think that I would like to use this system frequently
    a. Scale 1-5
2. I found the system unnecessarily complex
    a. Scale 1-5
3. I thought the system was easy to use
    a. Scale 1-5
4. I think that I would need the support of a technical person to be able to use this system
    a. Scale 1-5
5. I found the various functions in this system were well integrated
    a. Scale 1-5
6. I thought there was too much inconsistency in this system
    a. Scale 1-5
7. I would imagine that most people would learn to use this system very quickly
    a. Scale 1-5
8. I found the system very cumbersome to use
    a. Scale 1-5

9. I felt very confident using the system
    a. Scale 1-5
10. I needed to learn a lot of things before I could get going with this system
    a. Scale 1-5

Section 2 – Personal Motivation to Use Technology for Self-Management [mHealth Technology Engagement Index (mTEI)]

1. I should be able to pick and choose my treatment
    a. Scale 1-5
2. I should be able to make choices that improve my care
    a. Scale 1-5
3. I should be able to make choices that positively impact my care
    a. Scale 1-5
4. I should feel competent about my healthcare
    a. Scale 1-5
5. I should be able to better manage my health
    a. Scale 1-5
6. I should feel more knowledgeable about my health
    a. Scale 1-5
7. I should feel competent explaining my health to my healthcare provider
    a. Scale 1-5
8. I should feel like I can ask the right questions to my healthcare provider
    a. Scale 1-5
9. I should be able to connect with my healthcare provider
    a. Scale 1-5
10. I should have better interactions with my healthcare provider
    a. Scale 1-5
11. I should be able to connect to healthcare services I need
    a. Scale 1-5
12. I should feel closer to peers experiencing my health concern
    a. Scale 1-5
13. I should be able to set long-term goals
    a. Scale 1-5
14. I should be able to set short-term goals
    a. Scale 1-5
15. I should be able to show my healthcare provider my progress
    a. Scale 1-5
16. I should be able to visualize my progress
    a. Scale 1-5

Section 3 – App quality and perceived impact [based on User version of Mobile Application Rating Scale (uMARS)]

1. Entertainment: the system was fun/entertaining to use
    a. Scale 1-5

2. Interest: the system was interesting to use, it presented its information in an interesting way compared to other similar apps
    a. Scale 1-5
3. Customisation: the system allowed me to customise the settings and preferences that I would like to (e.g. sound, content and notifications)
    a. Scale 1-5
4. Interactivity: the system allows user input, provides feedback, contains prompts (reminders, sharing options, notifications, etc.)
    a. Scale 1-5
5. Target group: the system content (visuals, language, design) was appropriate for the target audience
    a. Scale 1-5
6. Performance: the system features (functions) and components (buttons/menus) worked accurately/fast
    a. Scale 1-5
7. Ease of use: it was easy to learn how to use the system; the menu labels, icons and instructions were clear
    a. Scale 1-5
8. Navigation: moving between screens made sense
    a. Scale 1-5
9. Gestural design: the taps/swipes/pinches/scrolls made sense; they were consistent across all components/screens
    a. Scale 1-5
10. Layout: the arrangement and size of buttons, icons, menus, and content on the screen were appropriate
    a. Scale 1-5
11. Graphics: there were high quality/resolution of graphics used for buttons, icons, menus and content
    a. Scale 1-5
12. Visual appeal: the system looks good
    a. Scale 1-5
13. Quality of information: the system content was correct, well written, and relevant to the goal/topic of the system
    a. Scale 1-5
14. Quantity of information: the information within the system was comprehensive but concise
    a. Scale 1-5
15. Visual information: the visual explanation of concepts through charts/graphs/images/videos, etc. was clear, logical, correct
    a. Scale 1-5
16. Credibility of source: the information within the system seemed to come from a credible source
    a. Scale 1-5
17. I would recommend this system to people who might benefit from it
    a. Scale 1-5
18. How many times do you think you would use this system in the next 12 months if it was relevant to you?
    a. None
    b. 1-2

    c. 3-10
    d. 10-50
    e. >50

19. I would pay for this system
    a. Scale 1 - 5
20. What is your overall (star) rating of the system (out of 5)?
    a. Number 1 – 5
21. Awareness – This study has increased my awareness of the importance of addressing the health behaviour
    a. Scale 1 - 5
22. Knowledge – This study has increased my knowledge/understanding of the health behaviour
    a. Scale 1 - 5
23. Attitudes – The study has changed my attitudes toward improving this health behaviour
    a. Scale 1 - 5
24. Intention to change – The study has increased my intentions/motivation to address this health behaviour
    a. Scale 1 - 5
25. Help seeking – This app would encourage me to seek further help to address the health behaviour (if I needed it)
    a. Scale 1 - 5
26. Behaviour change – Use of this app will increase/decrease the health behaviour
    a. Scale 1 - 5

Section 4 – General

1. I would pay for the FindAir ONE (RRP 59.00 EUR per year)
    a. Scale 1-5
2. I would pay for the Smart Asthma smart peak flow meter (RRP 59.60 GBP)
    a. Scale 1-5
3. I would pay for the Mi Band 3 smartwatch (RRP 25.00 GBP)
    a. Scale 1-5
4. What features / information would you most want to see in an asthma management system?
    a. Free text
5. Any other comment
    a. Free text

Brooke, John. "Sus: a “quick and dirty usability." *Usability evaluation in industry* 189 (1996).

Dewar, Alexis R., Tyler P. Bull, Donna M. Malvey, and James L. Szalma. "Developing a measure of engagement with telehealth systems: The mHealth Technology Engagement Index." *Journal of telemedicine and telecare* 23, no. 2 (2017): 248-255.

Stoyanov, Stoyan R., Leanne Hides, David J. Kavanagh, and Hollie Wilson. "Development and validation of the user version of the Mobile Application Rating Scale (uMARS)." *JMIR mHealth and uHealth* 4, no. 2 (2016): e72.
